# Up-Regulation of PARP1 Expression Significantly Correlated with Poor Survival in Mucosal Melanomas

**DOI:** 10.3390/cells9051135

**Published:** 2020-05-05

**Authors:** Piotr Donizy, Cheng-Lin Wu, Jason Mull, Masakazu Fujimoto, Agata Chłopik, Yan Peng, Sara C. Shalin, M. Angelica Selim, Susana Puig, Maria-Teresa Fernandez-Figueras, Christopher R. Shea, Wojciech Biernat, Janusz Ryś, Andrzej Marszalek, Mai P. Hoang

**Affiliations:** 1 Department of Pathomorphology and Oncological Cytology, Wroclaw Medical University, 50-556 Wroclaw, Poland; piotrdonizy@wp.pl; 2 Department of Pathology, National Cheng Kung University Hospital, College of Medicine, National Cheng Kung University, Tainan 70403, Taiwan; wujl.towalkwithwings@gmail.com; 3 Department of Pathology, University of Texas Southwestern Medical Center, Dallas, TX 75390, USA; Jason.a.mull@gmail.com (J.M.); Yan.peng@UTSouthwestern.edu (Y.P.); 4 Department of Pathology, Wakayama Medical University, Wakayama 641-8510, Japan; fujimasa2014@gmail.com; 5 Department of Pathology, Poznan University Medical Sciences and Greater Poland Cancer Center, 61-701 Poznan, Poland; agata.chlopik@gmail.com (A.C.); amars@ump.edu.pl (A.M.); 6 Department of Pathology, University of Arkansas for Medical Sciences, Little Rock, AR 72205, USA; SCShalin@uams.edu; 7 Department of Pathology, Duke University Medical Center, Durham, NC 27710, USA; Angelica.selim@duke.edu; 8 Department of Dermatology, Melanoma Unit, Hospital Clínic de Barcelona, IDIBAPS, and Centre of Biomedical Research on Rare Diseases (CIBERER), ISCIII, 08036 Barcelona, Spain; susipuig@gmail.com; 9 Department of Pathology, Hospital Universitari Germans Trias i Pujol, Hospital Universitari General de Catalunya, Grupo QuironSalud, Universitat Internacional de Catalunya, 08916 Barcelona, Spain; maiteffig@gmail.com; 10 Department of Medicine, Section of Dermatology University of Chicago, IL 60637, USA; cshea@medicine.bsd.uchicago.edu; 11 Department of Pathology, Medical University of Gdansk, 80-210 Gdansk, Poland; biernat@gumed.edu.pl; 12 Department of Tumor Pathology, Maria Sklodowska-Curie National Research Institute of Oncology, 31-115 Crakow Branch, Poland; z5rys@cyf-kr.edu.pl; 13 Department of Pathology, Massachusetts General Hospital and Harvard Medical School, Boston, MA 02114, USA

**Keywords:** mucosal melanoma, vulvar melanoma, sinonasal melanoma, anorectal melanoma, PARP, IDO1, prognosis, neoplasia

## Abstract

Introduction: Mucosal melanoma is rare and associated with poorer prognosis in comparison to conventional melanoma subtypes. Little is known about the prognostic significance as well as possible associations between PARP1 and immunologic response in mucosal melanoma. Methods: PARP1, PD-L1 and IDO1 immunostains were performed on 192 mucosal melanomas including 86 vulvar, 89 sinonasal, and 17 anorectal melanomas. Results: By Kaplan–Meier analyses, high PARP1 expression correlated with worse overall and melanoma-specific survival (log-rank *p* values = 0.026 and 0.047, respectively). Tumors with combined PARP1 and IDO1 high expression correlated with worse overall and melanoma-specific survival (*p* = 0.015, 0.0034 respectively). By multivariate analyses, high PARP1 expression remained a predictor of worse survival independent of stage. By Fisher’s exact test, high PARP1 expression correlated with highly mitogenic tumors (*p* = 0.02). High tumoral PD-L1 and IDO1 expression were associated with ulcerated primary tumors (*p* = 0.019, 0.0019, respectively). By linear regression analyses, correlations between PARP1 expression versus IDO1 expression (*p* = 0.0001) and mitotic index (*p* = 0.0052) were observed. Conclusion: Increased expression of PARP1 is an independent negative prognostic marker in mucosal melanomas. The association between PARP1 and IDO1 and their combined adverse prognostic role raise the potential of combined therapy in mucosal melanoma.

## 1. Introduction

Mucosal melanomas encompass primary melanomas of the head and neck region (sinonasal and oral melanoma), female genital tract (vulvar and vaginal melanoma), anorectal melanoma, upper gastrointestinal tract (esophagus, stomach, intestine), and urinary tract (urethra, urinary bladder) [1]. Mucosal melanomas are rare and constitute approximately 1.4% of all melanomas; however, they are biologically aggressive, often presenting at later stage due to an internal tumor location that is not visible or accessible, and are twice as lethal in comparison to cutaneous melanomas [2]. The five-year survival of mucosal melanoma is 78% versus 89% in cutaneous melanoma [3]. Mucosal melanomas are typically resistant to conventional therapies, namely surgery and postoperative radiotherapy [4]. Although immune-checkpoint inhibitor therapy has been shown to significantly improve overall survival (OS) in mucosal melanoma, durable response has not been uniformly observed for all patients [5].

PolyADP-ribosylation, which is catalyzed mainly by poly (ADP-ribose) polymerase 1 (PARP1), is a major post-translational modification that facilitates deoxyribonucleic acid (DNA) repair and chromatin remodeling [6]. Although PARP1 is crucial for DNA damage repairs, this enzyme is also involved in regulation of mitosis and immunologic response [7,8,9,10]. PARP inhibitors (PARPi), which are used either alone or in combination with DNA damage agents, may cause a G2/M mitotic arrest, apoptosis, or mitotic catastrophe [11,12]. Previous studies suggest that PARP-1 overexpression is a potential novel biomarker of aggressive clinical behavior in cutaneous malignant melanoma [13], but there are no studies focused on the prognostic significance of PARP1 expression in mucosal melanoma patients.

Impaired DNA repair and the associated genomic instability is a potential effect of abnormal activity of PARP1 in neoplastic cells, driving mutagenicity, which increases neoantigen load and tumoral immunogenicity [14]. Little is known about the possible associations between PARP1 and immunologic response in mucosal melanoma. The potential interaction between PARP1, IDO1, and PD-L1 has not been studied in a large cohort of mucosal melanomas. Understanding the clinical and pathologic features associated with expression of these immune markers in mucosal melanoma may provide insights into effective strategies for combination therapy.

Expression of programmed cell death ligand 1 (PD-L1) on tumoral cells contributes to cancer immune evasion by interacting with PD1 on the surface of the T lymphocytes, resulting in inhibition of the CD8+ cytotoxic T-cell response. Indoleamine 2,3-dioxygenase 1 (IDO1) is an immunosuppressing enzyme that induces T cell dysfunction and apoptosis through the depletion of tryptophan and the generation of kynurenine [15]. IDO1 is expressed in tumoral cells and adaptive immune response cells, and its reactivity correlates with a shorter survival in several cancers [16,17]. Due to its deregulated activity in human cancers, IDO1 is a possible target for combination therapies using IDO1 inhibitors (several in preclinical evaluation) and PD1/PD-L1 axis inhibitors [18]. Due to the effect of IDO1-mediated NAD+ production, and the fact that NAD+ is necessary for PARP1 activity [19], these two proteins are promising candidates for combined therapy (PARP1 and IDO1 inhibitors) as a hypothetical double-drug therapy covering multiple oncogenic pathways.

In the current study, we investigate the prognostic role of PARP1, PD-L1, and IDO1 protein expression and explore the possible associations between tumoral PARP1, PD-L1, and IDO1 expression in a series of mucosal melanomas.

## 2. Materials and Methods

The study was approved by institutional review boards (protocol# 2011P001665, approval date: July 2, 2019). One hundred and ninety two tumors from 192 patients which were diagnosed as primary vulvar (86), sinonasal (89) and anorectal melanomas (17) with available archival materials between 1989 and 2018 were retrieved from the pathology archives of 11 clinical institutions in Japan, Poland, Spain, Taiwan, and United States.

### 2.1. Clinical Findings and Histologic Features

The histopathologic diagnoses and features were assessed by the contributing pathologists and confirmed by the corresponding author (M.P.H.). The following data were extracted from medical records: age of the patients, lesion site, date of biopsy, and disease status over time and at last follow-up (recurrence, metastasis).

### 2.2. Immunohistochemistry

Immunohistochemical studies were performed on 5-micrometer-thick tissue sections with primary antibodies against PARP1 (clone: sc-74470 (B10), dilution: 1:50, Santa Cruz Biotechnology, Santa Cruz, CA, USA), PD-L1 (E1L3N, 1:200, Cell Signaling Technology, Danvers, MA), and IDO1 (1F8.2, 1:400, Millipore, Burlington, MA) for 169, 174, and 159 primary tumors, respectively. For PARP1 and IDO1, the slides underwent heat-induced epitope retrieval (HIER) with EnVision Target Retrieval Solution (Agilent DAKO, Santa Clara, CA) in a 30-min incubation at 97 °C in PT Link Pre-Treatment Module for Tissue Specimens (DAKO). Automated immunohistochemical staining was performed in Autostainer Link 48 (DAKO) and Liquid Permanent Red (Agilent DAKO) was utilized as a detection system. For PD-L1, the slides were incubated with a ready to use EDTA based Ph 9.0 epitope retrieval solution (Leica Microsystems, Bannockburn, IL, USA) for 20 min. Immunostaining was performed in Bond 3 automated immunostainer (Leica Microsystems). Human placenta and cutaneous melanoma were used as positive controls. The percentage of tumor cells with 5% or greater PD-L1 membranous staining of any intensity was considered positive. Scoring of IDO1 and PARP1 immunostains was done using the H-score ((percentage at 1+) × 1 + (percentage at 2+) × 2 + (percentage at 3+) × 3).

### 2.3. Statistical Analysis

The statistical associations between expression of PARP1, PD-L1, IDO1, and clinicopathologic features (patient’s age, stage, mitotic index, ulceration, lymphovascular invasion, and perineural invasion) were evaluated by Fisher’s exact tests. Time of death was equated to melanoma-related death in those who have had disease progression. Melanoma-specific survival (MSS) and overall survival (OS) were defined as the number of months from initial diagnosis to patient’s death related to melanoma and by any cause, respectively. Kaplan–Meier plots and log-rank tests were done to visually assess the differences in MSS and OS between subgroups. Correlations between number of mitoses and PD-L1 percent expression versus PARP1 and IDO1 H-scores were evaluated using linear regression analyses. Multivariate analyses were performed with the Cox proportional hazards model by including all statistically significant covariates from univariate Cox models. The proportionality assumptions of the Cox models were tested. All analyses and plots were performed using the R statistical package [20]. A two-tailed p-value of less than or equal to 0.05 was considered to be statistically significant.

## 3. Results

One hundred and ninety two patients were included in the study. The age of the patients ranged from 20 to 91 years (median, 65 years). The range of follow-up for all patients was 0 to 233 months (median, 23 months). Progression (local recurrence and/or metastasis) developed in 126/192 (66%) patients. Death was documented in 107/192 (56%) patients. Due to incomplete data such as tumor size and tumor thickness in cases such as sinonasal melanomas, the patients were divided into stage I/II versus stage III/IV to reflect whether metastasis was documented at time of diagnosis. There were 158 patients with stage I/II, 27 with stage III/IV, and 7 without known stage.

**Figure 1:**
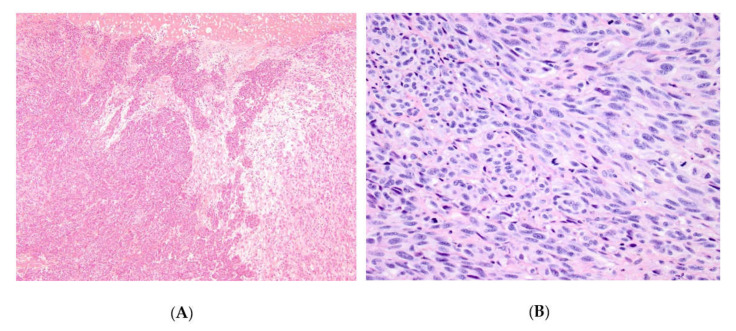
Ulceration in an anorectal melanoma (**A**) and prominent mitotic figures in another invasive vulvar melanoma (**B**).

Ulceration (Figure 1A), perineural invasion and lymphovascular invasion were noted in 143/192 (74%), 27/192 (14%), and 36/192 (19%) cases, respectively. The number of mitoses per squared millimeters ranged from 0 to 100 (Figure 1B).

### 3.1. Expression of PARP1, IDO1, and PD-L1 in Mucosal Melanoma Cells.

Due to the multicenter nature of the study, archival materials were not uniformly available for the studied cases. PARP1, PD-L1, and IDO1 were performed on 169, 174, and 159 primary tumors, respectively. All 3 immunostains were performed on 137 cases; PARP1 and IDO1 on 20 cases; PARP1 and PD-L1 on 12 cases; PD-L1 and IDO1 on 2 cases; and PD-L1 on 23 cases.

**Figure 2:**
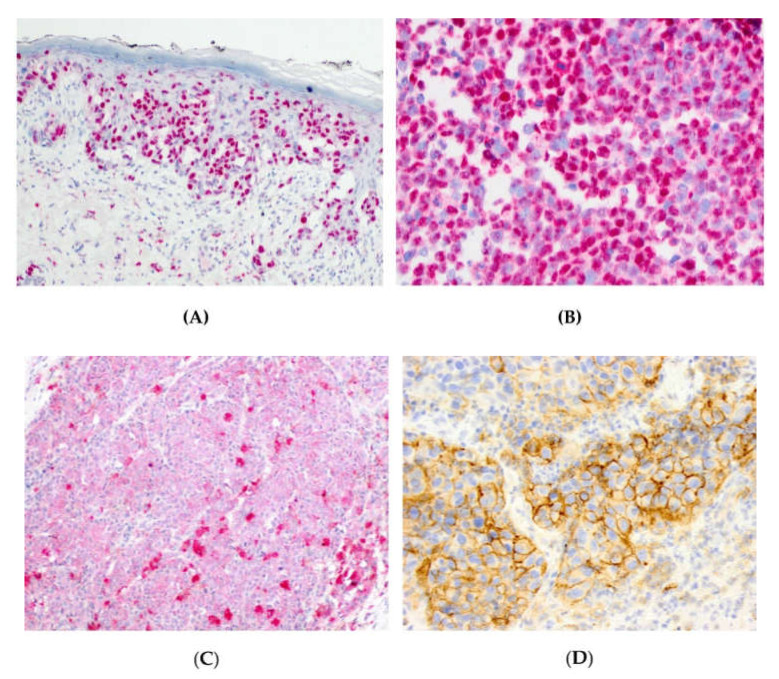
Nuclear PARP1 expression within an in situ (**A**) and invasive (**B**) component of a vulvar melanoma. Cytoplasmic IDO1 expression in anorectal melanoma (**C**). Membranous PD-L1 expression in a sinonasal melanoma (**D**).

Nuclear PARP1 H-scores ranged from 0 to 290, and H-score equal or above 200 was noted in 72/169 (43%) cases (Figure 2A,B). Membranous PD-L1 expression was seen from 1% to 70% with median of 5%. IDO1 H-scores ranged from 25 to 290, and H-score equal or above 180 was noted in 79/161 (49%) cases (Figure 2C). PD-L1 expression ≥5% was seen in 47/174 (27%) cases (Figure 2D). PD-L1 expression was noted in 24%, 40%, and 12% of vulvar (19/78), sinonasal (36/89), and anorectal (2/17) melanomas, respectively.

### 3.2. Correlations between PARP1, IDO1, and PD-L1 Expression and Clinicopathologic Variables

By Fisher’s exact test (Table 1), up-regulation of PARP1 in mucosal melanoma cells was significantly correlated with highly mitogenic tumors (*p* = 0.02). Low IDO1 expression in tumoral cells was correlated with decreased mitotic rate, but this correlation did not reach statistical significance (*p* = 0.067). High expression of tumoral PD-L1 and IDO1 were associated with ulcerated primary tumors (*p* = 0.019, and *p* = 0.0011, respectively). Overexpression of IDO1 in neoplastic cells correlated with absence of lymphangioinvasion (*p* = 0.032). Co-expression of PD-L1 and IDO1 was observed, but this association did not reach statistical significance (*p* = 0.061).

**Figure 3:**
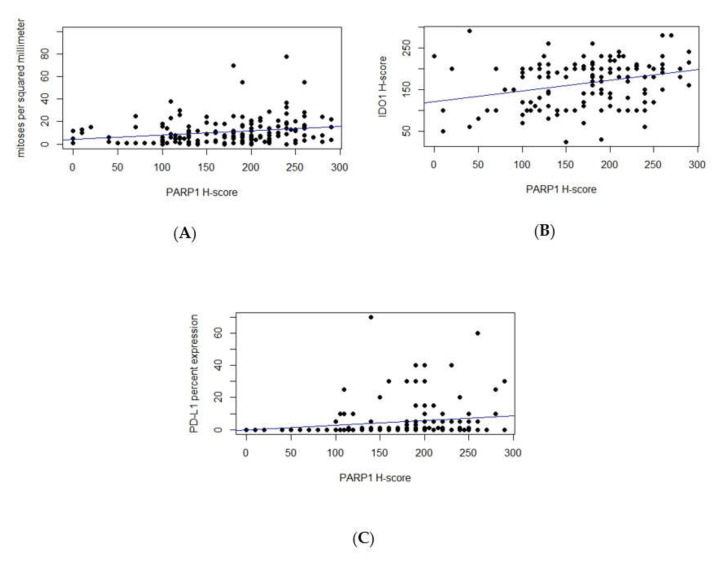
Using linear regression analyses, correlations were observed between PARP1 H-scores and mitotic index (*p* = 0.0052) (**A**), IDO1 H-scores (*p* = 0.0001) (**B**), and PD-L1 percent expression (*p* = 0.04) (**C**).

By linear regression analyses, increased number of mitoses measured per 1 mm2 was correlated with overexpression of PARP1 in nuclei of mucosal melanoma cells (*p* = 0.0052) (Figure 3A) but not to IDO1 or PD-L1 expression. Correlations between PARP1 overexpression in melanoma cells and presence of tumoral PD-L1 and IDO1 were observed (*p* = 0.04, and *p* = 0.0001, respectively) (Figure 3B,C).

### 3.3. Survival Analyses of PARP1, IDO1, and PD-L1 Expression in Mucosal Melanoma Patients

**Figure 4:**
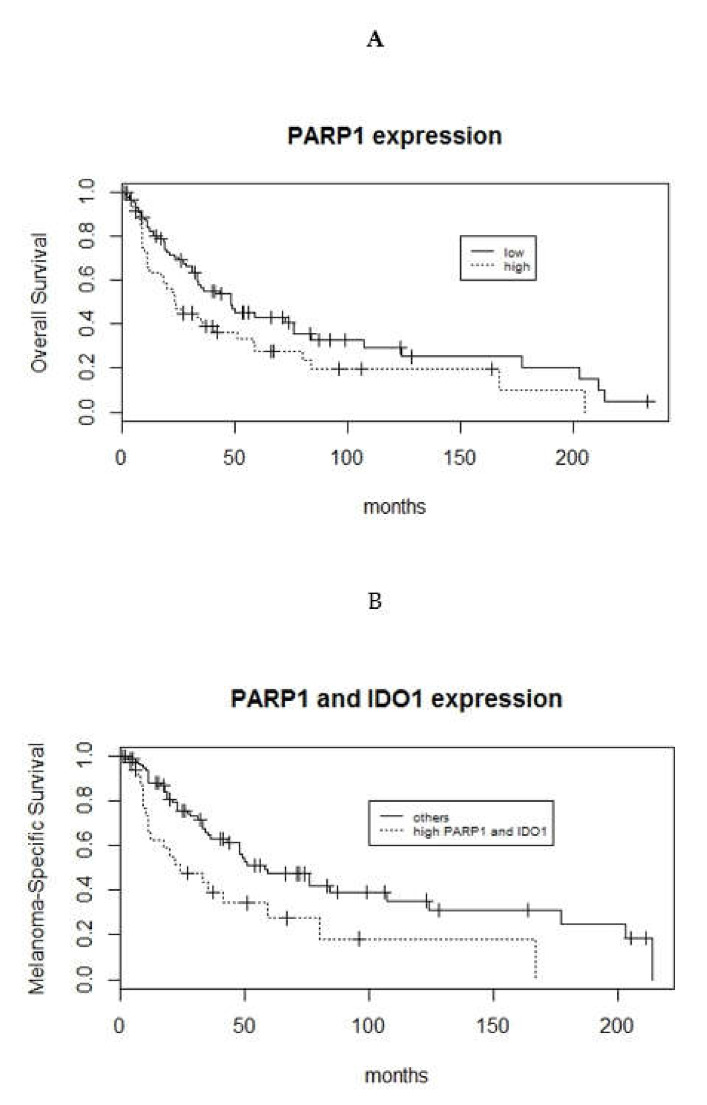
Kaplan–Meier curves illustrate the associations between high PARP1 expression and worse overall survival (log-rank *p*-value = 0.026) (**A**), and high PARP1 and high IDO1 expression and worse MSS (log-rank *p*-values = 0.0034) (**B**).

There was significant correlation between high PARP1 expression (H-score >200) and worse survival for both OS and MSS (log-rank *p*-value = 0.026 and 0.047, respectively) (Figure 4A). Tumors expressing both high PARP1 and high IDO1 H-scores correlated with worse OS and MSS (*p*-values = 0.015, 0.0034, respectively) versus the remainder (Figure 4B). There was no correlation observed between survival and tumoral IDO1 expression, PD-L1 expression, combined IDO1 and PD-L1 expression, and combined PARP1 and PD-L1 expression.

By univariate analyses (Table 2), the presence of ulceration in primary tumors and high PARP1 expression correlated with worse OS (*p* = 0.029, 0.027, respectively) and MSS (*p* = 0.035, 0.049, respectively). Higher stage (stages 3 and 4) correlated with worse MSS (*p* = 0.0062). There was no correlation observed between survival and tumoral IDO1 expression, PD-L1 expression, combined IDO1 and PD-L1 expression, and combined PARP1 and PD-L1 expression.

In multivariate analyses (Table 3), high PARP1 expression remained as an independent predictor of worse OS (*p* = 0.047). High PARP1 expression and higher stage remained as independent predictors of worse MSS (*p* = 0.04 and 0.0051, respectively). High PARP1 and IDO1 remained as independent predictors of worse OS and MSS (*p* = 0.017 and 0.0043, respectively).

## 4. Discussion

Melanomas originating from mucosal surfaces are rare and aggressive diseases associated with high rate of local recurrence and distant metastases. The poor prognosis is likely attributed to delay in diagnosis due to body location. In a series of 444 mucosal melanomas from a European population [21], head and neck location, male gender, advanced tumor stage, nodal disease, and incomplete resection status were independent risk factors for disease progression. Similarly, we observed stage to be an independent predictor of melanoma-specific survival.

PARP1 is a crucial mitotic-related protein. It has been found that PARP1 binds to proto-oncogenes, such as PDGFRB, EGFR/HER1, ERBB2/HER2, c-Src/CSK, SYK, Bruton’s tyrosine kinase, Abl2, MAP3K13, CDK18, and c-Mycs during mitosis in malignant cells [9,21]. Furthermore, PARP1 specifically bookmarks genes determined by NFATC2 (nuclear factor of activated T cells 2), a transcriptional factor which is a crucial regulator of oncogenic transformation [9,22,23]. The decrease in PARP1 protein levels promotes cell cycle arrest at prophase, and interaction between PARP1 and the mitotic checkpoint gene CHFR is crucial for the regulation of mitotic activity [24].

PARP1 overexpression has been reported to be a potential marker of aggressive clinical behavior in cutaneous malignant melanoma [13]. In the current study of mucosal melanoma, PARP1 overexpression is an independent poor prognostic marker. In addition, PARP1 overexpression significantly correlated with high mitotic activity in primary mucosal melanoma, which confirms the important role of PARP1 in regulation of mitosis. Previous in vitro and clinical studies revealed that PARP1 inhibition in highly mitogenic tumors reduced proliferation rate [25] and caused death of neoplastic cells in the mechanism of mitotic catastrophe [12]. In acute myeloid leukemia, inhibition of PARP1 induced neoplastic cell apoptosis and arrested cell cycle in G2/M phase [26]. In line with our observation, Jacot et al. [27] revealed that increased activity of PARP1 in breast cancer patients was significantly linked to high number of mitotic count. Moreover, Jacot et al. [27] did not observe any significant associations between activity of PARP1 and crucial clinical parameters such as TNM classification, steroid hormone receptors, HER2 status, and molecular profiles. Results similar to ours were obtained by Bertucci et al. [28] in 1432 primary soft tissue sarcomas. PARP1 overexpression measured by mRNA level was significantly correlated with high pathological FNCLCC (Fédération Nationale des Centres de Lutte le Cancer) grade, where mitotic count is an important parameter. Due to the significant correlation between high PARP1 expression, poor prognosis, and enhanced mitotic activity, potential inhibition of PAPR1 in mucosal melanoma patients could be beneficial.

The immunomodulatory roles of PARP-1 is complex with tissue-dependent multifactorial regulation pathways [29]. On one hand, PARP1 promotes inflammation by a several molecular mechanisms such as by positively regulating the pro-inflammatory NF-κB transcription factors [30] or suppressing the functions of Tregs due to PARylation of FoxP3 [31]. On the other hand, in acute myeloid leukemia PARP1 is involved in repression of NK cell activity by downregulation of NK cell-activating receptor ligands [32,33].

Besides immunologic-dependent procarcinogenic mechanisms mediated by IDO1, which are predominantly based on inhibition of T cell activation and facilitating immune evasion of tumor cells [34,35,36], little is known about immune-independent functions of IDO1 in carcinogenesis. In our study, we observed that low IDO1 expression in tumor cells was linked to decreased mitotic rate, but this correlation did not reach statistical significance. This result is in line with in vitro observation, where IDO1-mediated depletion of tryptophan induced cell cycle arrest in T cells at G1 [37], and correlated with accumulation of tumor cells in G1 and depletion of cells in G2/M phase [38]. Interestingly, Maleki Vareki et al. [38] showed that antisense-mediated reduction of IDO1, alone and in combination with antisense to the DNA repair protein BRCA2, sensitizes human lung cancer cells to olaparib and cisplatin.

Due to the fact that possible inhibition of IDO1 by specific inhibitors would decrease NAD+ in human cancer cells and that NAD+ is crucial for PARP1 activity, we hypothesize that double-hit therapy of IDO1 and PARP1 inhibitors would be a promising combination treatment. In our study, we observed a strong correlation between high PARP1 and IDO1 expressions in mucosal melanomas. In addition, combined high expression of both markers correlated with poor survival. These findings suggest that double inhibition of these proteins would be a potentially valuable immune-independent treatment modality.

Little is known about the possible interaction between PARP1 and PD-L1 in human cancers. There are no studies focused on this topic in mucosal melanoma. Theoretically, PARPi could increase the clinical sensitivity of tumors to immunotherapy by increasing mutation burden and generating plenty of neoantigens [39]. In our study, we observed a significant correlation between high nuclear expression of PARP1 and enhanced immunoreactivity of PD-L1 in mucosal melanoma cells. This is in line with the current immunologic paradigm that impaired DNA repair (high PARP1 expression as its potential histopathologic surrogate) increases neoantigen load and tumoral immunogenicity (enhanced PD-L1 expression on tumor cells as an immunohistochemical equivalent of adoptive immune response). On the other hand, Ding et al. [40] observed an inverse correlation between PARP1 and PD-L1 in 55 ovarian cancer patients. This discrepancy could be explained by the different type of cancer analyzed in these studies.

Jiao et al. [41] revealed an upregulation of PD-L1 in breast cancer cells and animal models after using PARP inhibitor (PARPi, olaparib). Furthermore, PARPi-induced PD-L1 expression suppresses anticancer immunity, and blockade of PD-L1 potentiates PARPi. An in vitro preclinical study performed by Wang et al. [42] demonstrated synergistic antitumor activities of niraparib (a selective PARP1/2 inhibitor) with anti-PD-1 immune checkpoint inhibitors. This study indicated that niraparib treatment increases the activity of the type I (alpha) and type II (gamma) interferon pathways and increases the infiltration of CD8+ cells and CD4+ lymphocytes in tumors. The combined therapy of PARPi and anti-PD-L1 would be a new promising molecular-based therapy in mucosal melanoma patients.

Our study has several limitations. First, tumor thickness cannot be determined in the majority of mucosal melanomas especially sinonasal melanomas due to the fragmented nature of the specimens and tangential nature of the histologic sections. Second, we did not stratify risk based on gender since all patients with vulvar melanomas would be female. Lastly, treatment modality of our cases was not uniform due to its multicenter nature, which was necessary in order to accrue a series of rare melanoma subtypes.

## 5. Conclusions

Increased expression of PARP1 is an independent negative prognostic marker in mucosal melanomas. The association between PARP1 and IDO1 and their combined negative prognostic role raise the potential of combined therapy in mucosal melanoma. Further in vitro and clinical studies are warranted.

## Figures and Tables

**Table 1:** Summary of clinicopathologic variables versus PAPR1, PD-L1, and IDO1 expression.

	PARP1 N = 167			PD-L1 N = 174			IDO1 N = 159		
	High	Low	*p*-Value	High	Low	*p*-Value	High	Low	*p*-Value
**Age**									
>65 years	41	46	0.44	20	62	0.4	39	45	0.43
< = 65 years	42	38		28	64		40	35	
**Ulceration**									
Present	66	62	0.47	42	88	0.019*	70	53	0.0011*
Absent	17	22		6	38		9	27	
**Mitoses**									
>4 / mm^2^	49	34	0.02*	24	60	0.87	69	60	0.067
<= 4 / mm^2^	34	50		24	66		10	20	
**Lymphovascular Invasion**									
Present	18	13	0.33	9	21	0.82	22	11	0.032 *
Absent	65	71		38	105		57	69	
**Perineural invasion**									
Present	11	14	0.67	5	16	0.80	9	16	0.19
Absent	72	70		42	110		70	64	

* *p* < 0.05, statistically significant.

**Table 2:** Univariate Cox proportional hazards model.

	Overall Survival		Melanoma Specific Survival	
	Hazard Ratio	*p*-Value	Hazard Ratio	*p*-Value
Age	1.14	0.49	1.10	0.65
Stage (1–2 versus 3–4)	1.6	0.095	2.19	0.0062 *
Ulceration	1.69	0.029 *	1.78	0.035 *
Mitoses	1.47	0.12	1.54	0.13
Lymphovascular invasion	1.03	0.91	1.2	0.52
Perineural invasion	1.05	0.86	1.2	0.64
PARP1 expression	1.59	0.027 *	1.61	0.049 *
IDO1 expression	1.3	0.22	1.31	0.28
PD-L1 expression	0.75	0.23	0.71	0.21
PARP1 and IDO1 expression	1.77	0.017 *	2.14	0.0043 *
PARP1 and PD-L1 expression	1.35	0.28	1.37	0.33
IDO1 and PD-L1 expression	1.24	0.46	1.33	0.39

* *p* < 0.05, statistically significant; *p* < 0.09, approaching statistical significance.

**Table 3:** Multivariate Cox proportional hazards models.

	Overall Survival		Melanoma Specific Survival	
	Hazard Ratio	*p*-Value	Hazard Ratio	*p*-Value
PARP1 expression	1.53	0.047 *	1.68	0.04 *
Ulceration	1.31	0.30	1.48	0.2
Stage (1-2 versus 3-4)	-	-	2.43	0.0051 *
PARP1 and IDO1 expression	1.75	0.025 *	2.14	0.0069 *
Ulceration	1.13	0.66	1.25	0.5
Stage (1-2 versus 3-4)	-	-	1.96	0.039 *

* *p* < 0.05, statistically significant.

## References

[1] Chang A.E., Kamell L.H., Menck H.R. (1998). The National Cancer Data Base report on cutaneous and noncutaneous melanoma: A summary of 84,836 cases from the past decade. Cancer.

[2] Hahn H.M., Lee K.G., Choi W., Cheong S.H., Myung K.B., Hahn H.J. (2019). An updated review of mucosal melanoma: Survival meta-analysis. Mol. Clin. Oncol..

[3] Bishop K.D., Olszewski A.J. (2014). Epidemiology and survival outcomes of ocular and mucosal melanomas: A population-based analysis. Int. J. Cancer.

[4] Yao J.J., Zhang F., Zhang G.S., Deng X.W., Zhang W.J., Lawrence W.R., Zou L., Zhang X.S., Lu L.X. (2018). Efficacy and safety of primary surgery with postoperative radiotherapy in head and neck mucosal melanoma: A single-arm Phase II study. Cancer Manag. Res..

[5] Mignard C., Deschamps Huvier A., Gillibert A., Duval Modeste A.B., Dutriaux C., Khammari A., Avril M.F., Kramkimel N., Mortier L., Marcant P. (2018). Efficacy of immunotherapy in patients with metastatic mucosal or uveal melanoma. J. Oncol..

[6] Langelier M.F., Eisemann T., Riccio A.A., Pascal J.M. (2018). PARP family enzymes: Regulation and catalysis of the poly(ADP-ribose) posttranslational modification. Curr. Opin. Struct. Biol..

[7] Slade D. (2019). Mitotic functions of poly(ADP-ribose) polymerases. Biochem. Pharmacol..

[8] Iglesias P., Costoya J.A. (2014). The antimitotic potential of PARP inhibitors, an unexplored therapeutic alternative. Curr. Top Med. Chem..

[9] Lodhi N., Kossenkov A.V., Tulin A.V. (2014). Bookmarking promoters in mitotic chromatin: Poly(ADP-ribose)polymerase-1 as an epigenetic mark. Nucleic Acids Res..

[10] Perdoni F., Bottone M.G., Soldani C., Veneroni P., Alpini C., Pellicciari C., Scovassi A.I. (2009). Distribution of centromeric proteins and PARP-1 during mitosis and apoptosis. Ann. N. Y. Acad. Sci..

[11] Madison D.L., Stauffer D., Lundblad J.R. (2011). The PARP inhibitor PJ34 causes a PARP1-independent, p21 dependent mitotic arrest. DNA Repair (Amst.).

[12] Colicchia V., Petroni M., Guarguaglini G., Sardina F., Sahún-Roncero M., Carbonari M., Ricci B., Heil C., Capalbo C., Belardinilli F. (2017). PARP inhibitors enhance replication stress and cause mitotic catastrophe in MYCN-dependent neuroblastoma. Oncogene.

[13] Staibano S., Pepe S., Lo Muzio L., Somma P., Mascolo M., Argenziano G., Scalvenzi M., Salvatore G., Fabbrocini G., Molea G. (2005). Poly(adenosine diphosphate-ribose) polymerase 1 expression in malignant melanomas from photoexposed areas of the head and neck region. Hum. Pathol..

[14] Rosado M.M., Bennici E., Novelli F., Pioli C. (2013). Beyond DNA repair, the immunological role of PARP-1 and its siblings. Immunology.

[15] Munn D.H. (2011). Indoleamine 2,3-dioxygenase, Tregs and cancer. Curr. Med. Chem..

[16] Uyttenhove C., Pilotte L., Theate I., Stroobant V., Colau D., Parmentier N., Boon T., Van den Eynde B.J. (2003). Evidence for a tumoral immune resistance mechanism based on tryptophan degradation by indoleamine 2,3-dioxygenase. Nature Med..

[17] Brochez L., Chevolet I., Kruse V. (2017). The rationale of indoleamine 2,3-dioxygenase inhibition for cancer therapy. Eur. J. Cancer.

[18] Cheong J.E., Sun L. (2018). Targeting the IDO1/TDO2-KYN-AhR pathway for cancer immunotherapy—Challenges and opportunities. Trends Pharmacol. Sci..

[19] Khan J.A., Forouhar F., Tao X., Tong L. (2007). Nicotinamide adenine dinucleotide metabolism as an attractive target for drug discovery. Expert Opin. Ther. Targets.

[20] R Development Core Team (2019). R: A Language and Environment for Statistical Computing.

[21] Heppt M.V., Roesch A., Weide B., Gutzmer R., Meier F., Loquai C., Kähler K.C., Gesierich A., Meissner M., von Bubnoff D. (2017). Prognostic factors and treatment outcomes in 444 patients with mucosal melanoma. Eur. J. Cancer.

[22] Lodhi N., Ji Y., Tulin A. (2016). Mitotic bookmarking: Maintaining post-mitotic reprogramming of transcription reactivation. Curr. Mol. Biol. Rep..

[23] Tie X., Han S., Meng L., Wang Y., Wu A. (2013). NFAT1 is highly expressed in, and regulates the invasion of, glioblastoma multiforme cells. PLoS ONE.

[24] Kashima L., Idogawa M., Mita H., Shitashige M., Yamada T., Ogi K., Suzuki H., Toyota M., Ariga H., Sasaki Y. (2012). CHFR protein regulates mitotic checkpoint by targeting PARP-1 protein for ubiquitination and degradation. J. Biol. Chem..

[25] Quiles-Perez R., Muñoz-Gámez J.A., Ruiz-Extremera A., O’Valle F., Sanjuán-Nuñez L., Martín-Alvarez A.B., Martín-Oliva D., Caballero T., Muñoz de Rueda P., León J. (2010). Inhibition of poly adenosine diphosphate-ribose polymerase decreases hepatocellular carcinoma growth by modulation of tumor-related gene expression. Hepatology.

[26] Li X., Li C., Jin J., Wang J., Huang J., Ma Z., Huang X., He X., Zhou Y., Xu Y. (2018). High PARP-1 expression predicts poor survival in acute myeloid leukemia and PARP-1 inhibitor and SAHA-bendamustine hybrid inhibitor combination treatment synergistically enhances anti-tumor effects. EBioMedicine.

[27] Jacot W., Thezenas S., Senal R., Viglianti C., Laberenne A.C., Lopez-Crapez E., Bibeau F., Bleuse J.P., Romieu G., Lamy P.J. (2013). BRCA1 promoter hypermethylation, 53BP1 protein expression and PARP-1 activity as biomarkers of DNA repair deficit in breast cancer. BMC Cancer.

[28] Bertucci F., Finetti P., Monneur A., Perrot D., Chevreau C., Le Cesne A., Blay J.Y., Mir O., Birnbaum D. (2019). PARP1 expression in soft tissue sarcomas is a poor-prognosis factor and a new potential therapeutic target. Mol. Oncol..

[29] Yélamos J., Moreno-Lama L., Jimeno J., Ali S.O. (2020). Immunomodulatory Roles of PARP-1 and PARP-2: Impact on PARP-Centered Cancer Therapies. Cancers (Basel).

[30] Pazzaglia S., Pioli C. (2019). Multifaceted Role of PARP-1 in DNA Repair and Inflammation: Pathological and Therapeutic Implications in Cancer and Non-Cancer Diseases. Cells.

[31] Luo X., Nie J., Wang S., Chen Z., Chen W., Li D., Hu H., Li B. (2015). Poly(ADP-ribosyl)ation of FOXP3 Protein Mediated by PARP-1 Protein regulates the function of regulatory t cells. J. Biol. Chem..

[32] Heyman B., Jamieson C. (2019). To PARP or not to PARP? Toward sensitizing acute myeloid leukemia stem cells to immunotherapy. EMBO J..

[33] Paczulla A.M., Rothfelder K., Raffel S., Konantz M., Steinbacher J., Wang H., Tandler C., Mbarga M., Schaefer T., Falcone M. (2019). Absence of NKG2D ligands defines leukaemia stem cells and mediates their immune evasion. Nature.

[34] Munn D.H., Mellor A.L. (2013). Indoleamine 2,3 dioxygenase and metabolic control of immune responses. Trends Immunol..

[35] Godin-Ethier J., Hanafi L.A., Piccirillo C.A., Lapointe R. (2011). Indoleamine 2,3-dioxygenase expression in human cancers: Clinical and immunologic perspectives. Clin. Cancer Res..

[36] Zheng X., Koropatnick J., Li M., Zhang X., Ling F., Ren X., Hao X., Sun H., Vladau C., Franek J.A. (2006). Reinstalling antitumor immunity by inhibiting tumor derived immunosuppressive molecule IDO through RNA interference. J. Immunol..

[37] Munn D.H., Shafizadeh E., Attwood J.T., Bondarev I., Pashine A., Mellor A.L. (1999). Inhibition of T cell proliferation by macrophage tryptophan catabolism. J. Exp. Med..

[38] Maleki Vareki S., Rytelewski M., Figueredo R., Chen D., Ferguson P.J., Vincent M., Min W., Zheng X., Koropatnick J. (2014). Indoleamine 2,3-dioxygenase mediates immune-independent human tumor cell resistance to olaparib, gamma radiation, and cisplatin. Oncotarget.

[39] Li A., Yi M., Qin S., Chu Q., Luo S., Wu K. (2019). Prospects for combining immune checkpoint blockade with PARP inhibition. J. Hematol. Oncol..

[40] Ding L., Chen X., Xu X., Qian Y., Liang G., Yao F., Yao Z., Wu H., Zhang J., He Q. (2019). PARP1 Suppresses the Transcription of PD-L1 by Poly(ADP-Ribosyl)ating STAT3. Cancer Immunol. Res..

[41] Jiao S., Xia W., Yamaguchi H., Wei Y., Chen M.K., Hsu J.M., Hsu J.L., Yu W.H., Du Y., Lee H.H. (2017). PARP Inhibitor Upregulates PD-L1 Expression and Enhances Cancer-Associated Immunosuppression. Clin. Cancer Res..

[42] Wang Z., Sun K., Xiao Y., Feng B., Mikule K., Ma X., Feng N., Vellano C.P., Federico L., Marszalek J.R. (2019). Niraparib activates interferon signaling and potentiates anti-PD-1 antibody efficacy in tumor models. Sci. Rep..

